# A Yoga Strengthening Program Designed to Minimize the Knee Adduction Moment for Women with Knee Osteoarthritis: A Proof-Of-Principle Cohort Study

**DOI:** 10.1371/journal.pone.0136854

**Published:** 2015-09-14

**Authors:** Elora C. Brenneman, Alexander B. Kuntz, Emily G. Wiebenga, Monica R. Maly

**Affiliations:** 1 School of Rehabilitation Sciences, McMaster University, Hamilton, Ontario, Canada; 2 Department of Kinesiology, McMaster University, Hamilton, Ontario, Canada; Copenhagen University Hospital, Hvidovre, DENMARK

## Abstract

People with knee osteoarthritis may benefit from exercise prescriptions that minimize knee loads in the frontal plane. The primary objective of this study was to determine whether a novel 12-week strengthening program designed to minimize exposure to the knee adduction moment (KAM) could improve symptoms and knee strength in women with symptomatic knee osteoarthritis. A secondary objective was to determine whether the program could improve mobility and fitness, and decrease peak KAM during gait. The tertiary objective was to evaluate the biomechanical characteristics of this yoga program. In particular, we compared the peak KAM during gait with that during yoga postures at baseline. We also compared lower limb normalized mean electromyography (EMG) amplitudes during yoga postures between baseline and follow-up. Primary measures included self-reported pain and physical function (Knee injury and Osteoarthritis Outcome Score) and knee strength (extensor and flexor torques). Secondary measures included mobility (six-minute walk, 30-second chair stand, stair climbing), fitness (submaximal cycle ergometer test), and clinical gait analysis using motion capture synchronized with electromyography and force measurement. Also, KAM and normalized mean EMG amplitudes were collected during yoga postures. Forty-five women over age 50 with symptomatic knee osteoarthritis, consistent with the American College of Rheumatology criteria, enrolled in our 12-week (3 sessions per week) program. Data from 38 were analyzed (six drop-outs; one lost to co-intervention). Participants experienced reduced pain (mean improvement 10.1–20.1 normalized to 100; p<0.001), increased knee extensor strength (mean improvement 0.01 Nm/kg; p = 0.004), and increased flexor strength (mean improvement 0.01 Nm/kg; p = 0.001) at follow-up compared to baseline. Participants improved mobility on the six-minute walk (mean improvement 37.7 m; p<0.001) and 30-second chair stand (mean improvement 1.3; p = 0.006) at follow-up compared to baseline. Fitness and peak KAM during gait were unchanged between baseline and follow-up. Average KAM during the yoga postures were lower than that of normal gait. Normalized mean EMG amplitudes during yoga postures were up to 31.0% of maximum but did not change between baseline and follow-up. In this cohort study, the yoga-based strengthening postures that elicit low KAMs improved knee symptoms and strength in women with knee OA following a 12 week program (3 sessions per week). The program also improved mobility, but did not improve fitness or reduce peak KAM during gait. The KAM during the yoga postures were lower than that of normal gait. Overall, the proposed program may be useful in improving pain, strength, and mobility in women with knee osteoarthritis. Clinical efficacy needs to be assessed using a randomized controlled trial design.

**Trial Registration:**

ClinicalTrials.gov NCT02146105

## Introduction

The prevalence of knee osteoarthritis (OA) will increase in parallel with increasing age and obesity in Westernized countries [1]. While joint replacement is the gold standard treatment for knee OA, escalating demands in Canada, the United States, the United Kingdom, and Australia result in long wait times [2–5]. Implementing conservative treatments that improve symptoms, slow progression, and/or delay or prevent the need for surgery is critical. Systematic reviews and clinical practice guidelines confirm that strengthening exercise is the cornerstone of knee OA management [6–9].

Exercise reduces symptoms, comorbidity, and improves physical function [7–9] in people with knee OA. A meta-analysis of 32 studies of knee OA demonstrated a beneficial effect for pain (n = 3,616) and physical function (n = 3,719) [10]. Effect sizes for both were equivalent to those produced by pain medications [10]. Strength training also improved bone mass [11], aerobic capacity [12], and psychological health [13] in this population. Studies have implemented a variety of exercises, such as weight-bearing, non-weight-bearing, and neuromuscular exercises [6,14–16]. While there are numerous exercise programs for knee OA, the ideal type of strengthening for knee OA is unknown [17]. As a result, a need remains for studies investigating the ideal types and dosages for exercise prescription in knee OA [18]. It is interesting to note that no exercise program has been designed for knee OA based on biomechanical loads.

Mechanical loading is a probable contributor to cartilage degradation in knee OA [19–21] through mechanisms of cell death and alterations in the structure of cartilage [22,23]. The external knee adduction moment (KAM) reflects the relative medial-to-lateral distribution of force across the tibial plateau [24,25]. While the KAM is no substitute for contact forces in the medial knee, it is implicated in the progression of radiographic knee OA [22]. Over a 6 year period, a 25% increase in the peak KAM during gait increased the risk of radiographic progression by 6.46 times among 106 participants with knee OA [26]. More recent studies using magnetic resonance imaging provide similar findings over follow-up periods between 1 and 5 years [27,28]. We propose utilizing the KAM to identify appropriate exercise for people with knee OA. Given the growing body of evidence showing that exposure to large KAMs is associated with structural progression of knee OA, we developed an exercise intervention that minimized exposure to KAM. Our goal was to explore whether yoga postures that minimized the KAM could benefit clinical outcomes in people with symptomatic knee OA.

The literature has identified several principles that minimize the peak KAM during gait in knee OA: body centre of mass placed over the knee [29–31]; barefoot rather than shod weight-bearing activity [32]; external rotation of the foot [33,34]; low speed [35]; and low repetition [36]. While these strategies are specific to gait, the same principles likely apply to exercise. An exercise program for knee OA that takes advantage of this combination of biomechanics may reduce KAM and therefore abnormal loading at the knee joint. Some exercise programs focus on ideal lower extremity alignment, however these utilize footwear [15,37,38]. We identified that some, but not all, yoga postures combine elements of ideal lower extremity alignment, barefoot, foot alignment in external rotation, relatively slow speed, and minimal repetition. A few studies have investigated yoga as an appropriate intervention for knee OA, but these studies had limited sample sizes, limited exposure to exercise, and did not focus on lower limb biomechanics [39–41]. One study examined biomechanical loads associated with seven yoga exercises in healthy older adults [42]. This study found certain exercises (namely single leg stance poses) elicited KAMs greater or close to those experienced during normal gait. However, muscle activations in this previous paper were expressed relative to the maximum achieved during level walking rather than a maximal effort contraction, making it somewhat challenging to identify ideal strengthening exercises [42].

We previously identified postures that maximized quadriceps muscle activation to enable strengthening, while exposing the joint to relatively low magnitude KAMs [43]. Squat and lunge variations generated quadriceps muscle activations reaching 40% of the maximum voluntary isometric contraction (MVIC) and low magnitude KAMs (-0.29 to 0.18 Nm/kg), where negative values indicating a knee abduction moment; while positive values indicate a knee adduction moment [44]. These KAMs were below peak values experienced during walking, (0.3–0.5 Nm/kg) [45], a common exercise prescription for knee OA [46,47]. Single limb stance exercises produced higher KAMs and lower quadriceps muscle activations, suggesting these may not to be ideal for knee OA [42]. This previous work involved healthy young women; the generalizability of the actual KAM values to older women with knee OA would be unclear. Nonetheless, we expected that the patterns of exercises yielding high and low KAM exposures would be consistent between the samples. We used these data, along with previous literature, to design a strengthening program for knee OA. The impact of these low KAM postures on (1) symptoms and knee strength, (2) mobility and fitness, as well as (3) KAM and muscle activity, is unknown.

The purpose of this study was three-fold. The first objective was to identify whether a 12-week yoga-inspired strengthening program designed to minimize the KAM could improve symptoms and knee strength in women with symptomatic knee OA. Improvement in symptoms and strength are primary targets for conservative knee OA interventions [17,48]. A secondary objective was to evaluate whether the yoga program could improve mobility performance and cardiovascular fitness, and reduce the peak KAM during gait. Mobility performance and cardiovascular fitness are appropriate treatment targets for knee OA [49]. The third objective was to evaluate biomechanical outcomes. To ensure the yoga postures yielded a low KAM, we compared the average KAM during yoga postures with the peak KAM during gait at baseline. Also, we compared lower limb muscle normalized mean electromyography (EMG) amplitudes during yoga postures before and after the yoga program. We hypothesized that, at follow-up compared to baseline, participants would demonstrate (1) lower symptom scores and increased knee muscle strength; (2) improved mobility performance and cardiovascular fitness, and reduced peak KAM during gait; and (3) average KAM during the postures lower than that of normal gait; and normalized EMG amplitudes that decreased following the intervention.

## Materials and Methods

To establish proof-of-principle, measurements were recorded on a single-group before (baseline) and after (follow-up) intervention. Because this study was a single-group, blinding (of participants or assessors) was not conducted. We made a modification to the original protocol. We added a third objective to investigate whether postures produced a lower KAM to that of level walking; and to determine if normalized mean EMG amplitudes of lower limb muscles decreased as a result of the program. The postures investigated in the current study were evaluated in previous work in our laboratory with young healthy women and found that these postures produced a lower KAM to that of gait [43]. The added aim was to ensure the same results were observed in a knee OA population. As well, EMG was added to the protocol May 2014 (prior to baseline data collection) as there is limited information on the effect of a yoga-based exercise intervention on EMG muscle amplitude.

### Participants

**Fig 1:**
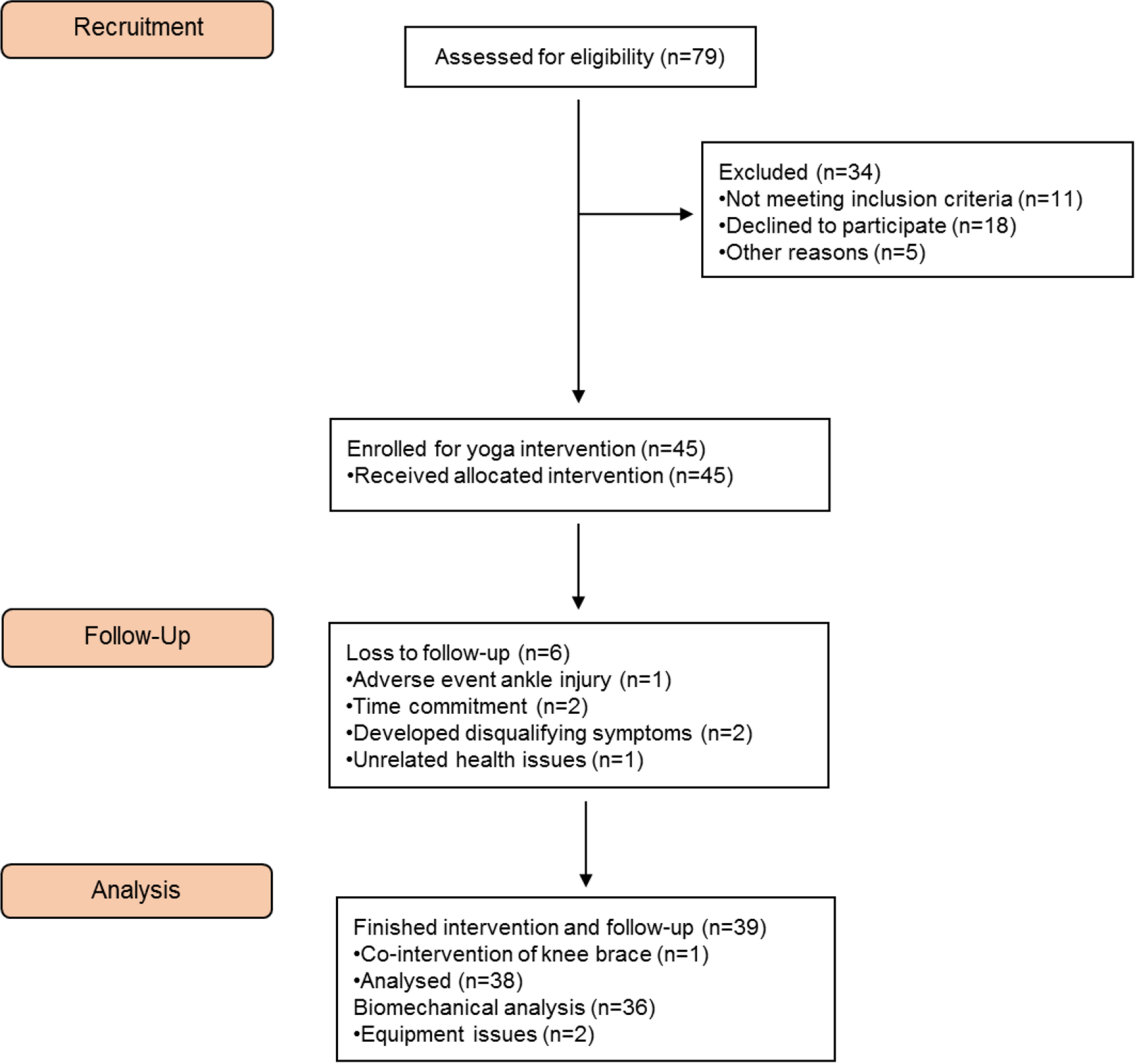
CONSORT diagram of participant flow through the recruitment, intervention allocation, and analysis of this study.

Participants were recruited through rheumatology, orthopaedic, and physical therapy clinics, as well as by word-of-mouth in the Hamilton, Ontario, Canada community. Community dwelling older women meeting the American College of Rheumatology (ACR) criteria for symptomatic knee OA participated. Screening was conducted by one rheumatologist and/or one trained research assistant. These clinical ACR criteria include being 50 years of age or older and answering “yes” on three of the following six criteria: having knee pain on most days of the week, crepitus, bony tenderness or enlargement, no warmth to the touch, or knee stiffness lasting longer than 30 minutes [50]. Exclusion criteria included alternate forms of arthritis (e.g., rheumatoid arthritis) or non-arthritic joint disease in the knee, previous knee surgeries, use an assistive walking aid on a regular basis, lower limb trauma within the past 3 months, ipsilateral ankle, knee or hip condition including pain under the knee cap, pregnancy, or any conditions that may have been exacerbated by the protocol (e.g., unstable angina). The participant flowchart is displayed in Fig 1.

### Ethics Statement

This study was approved by the Hamilton Integrated Research Ethics Board and all participants provided written, informed consent (REB#13–510).

### Study Setting

The study was conducted at the MacMobilize Laboratory at McMaster University, Hamilton, Ontario, Canada. The intervention was implemented at a private yoga studio in downtown Hamilton, Ontario, Canada.

### Intervention: Yoga for Lower Limb Muscle Strengthening

**Fig 2:**
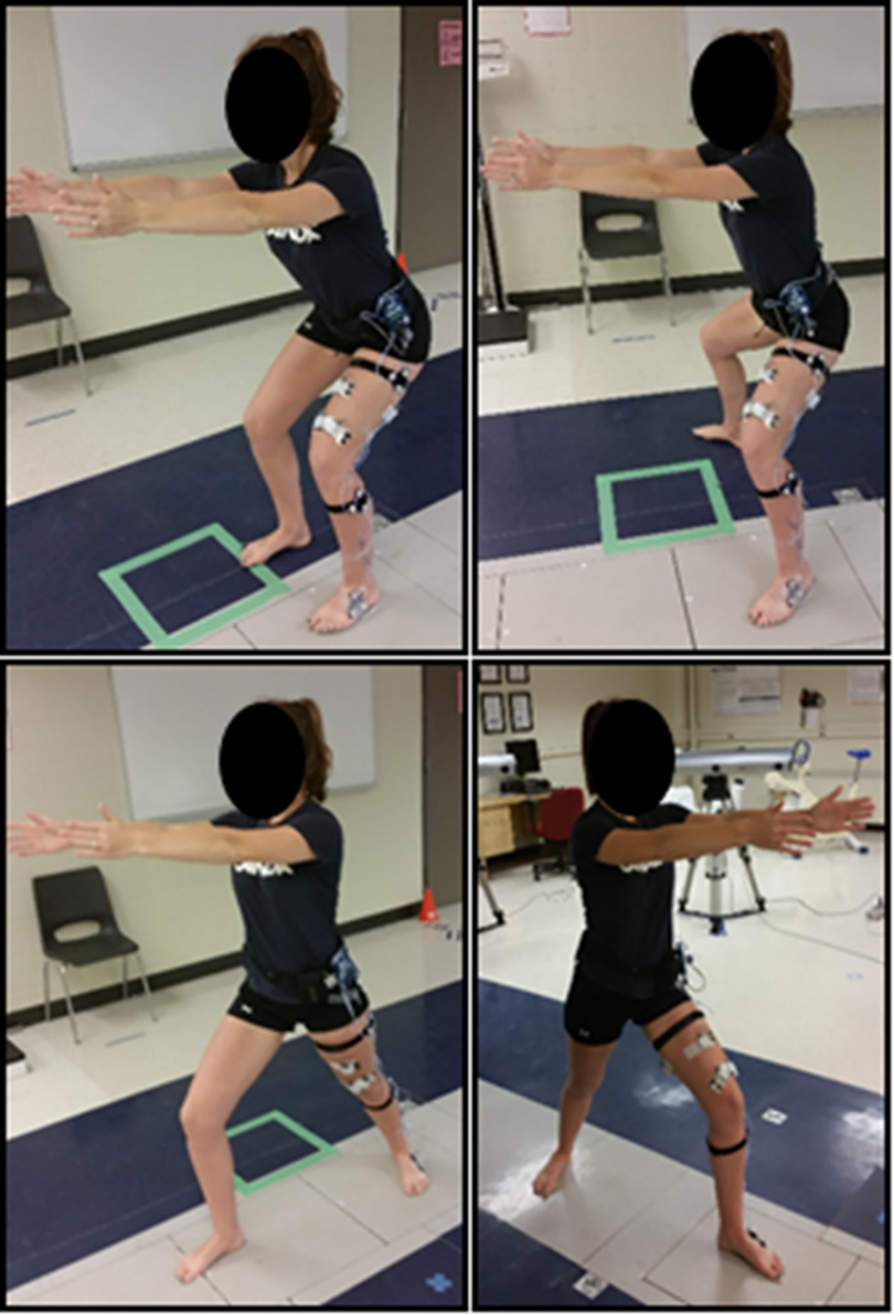
Yoga postures used in this lower limb strengthening program for knee osteoarthritis. The four postures investigated included the legs-together squat (top left), wide-legged squat (top right), and the lunge (bottom; the involved and uninvolved limb were analysed separately).

Participants completed a yoga-inspired, lower extremity strengthening program designed for knee OA. This supervised program was group-based and taught by a certified yoga instructor who was trained to deliver the strengthening program at a private yoga studio (De La Sol Yoga) in Hamilton, Ontario [44] (Fig 2, S1 Appendix). The yoga program focused on lower extremity strengthening and hip mobility using a variety of squats and lunges. Each one-hour class began with a warm-up of large range body movements against gravity. Then, the class focused on weight-bearing, isometric postures that emphasized quadriceps strengthening. These postures included squats (different feet positions); lunges (different feet and arm positions); supported lunges; and transitions from standing to and from standing, sitting, and lying. To balance the focus on quadriceps strengthening, postures including supine bridges and heel raises were incorporated to target the hamstrings and ankle plantarflexors respectively. Throughout the program, participants were educated and encouraged to position the knee over the 2^nd^ toe when weight-bearing in knee flexion. Exercise progressions are included in Table 1. Participants were encouraged to use any modification that allowed them to exert at a 7 on the Borg Perceived Exertion Scale (0–10 scale with 0 = no exertion and 10 = maximal exertion) [51]. If knee pain increased by 2/10 points from pre-exercise pain on a Visual Analog Scale (VAS) during any exercise, participants were instructed to ask for a modification to alleviate discomfort. Exercise modifications, which entailed alterations in knee range of motion and balance/weight-bearing support, were available for every posture. During the cool-down, participants completed stretches focusing on hip, knee and ankle musculature in supine.

**Table 1:** Levels 1 through 4 of the progressions in the yoga strengthening program. Please refer to S1 Appendix for further information on the exercises.

Yoga Posture	Level 1	Level 2	Level 3	Level 4
**Squat (legs together)**	Hands on hips, bend knees to 30° flexion	Hands on hips, bend knees to 60° flexion	Shoulders flexed to 90° with elbows straight, bend knees to 60° flexion	Arms over head, bend knees to 80° flexion, look up to ceiling for added challenge
**Squat (wide-legged)**	Hands on hips, bend knees to 30° flexion	Hands on hips, bend knees to 60° flexion	Shoulders flexed to 90° with elbows straight, bend knees to 60° flexion	Arms over head, bend knees to 80° flexion, look up to ceiling for added challenge
**Supported lunge**	Hands on hips, the hip of the trail leg is in neutral (no flexion, no extension)	Hands on hips, the hip of the trail leg is in extension	Shoulders flexed to 90° with elbows straight, the hip of the trail leg is in extension	Arms over head, the hip of the trail leg is in extension
**Lunge (lateral trunk)**	Elbow on knee of the lead leg, place other hand on the hip	Elbow on knee of the leading leg, flex other shoulder to 180°	Hand beside the foot instep of the lead leg, flex other shoulder to 180°	Hand beside lateral side of foot of the lead leg, flex other shoulder to 180°
**Lunge (upright trunk 1)**	Hands on hips	Shoulders flexed to 90° with elbows straight	Arms over head	Arms over head, look up to the ceiling for added challenge
**Lunge (upright trunk 2)**	Hands on hips	Arms abducted to 90° with elbows straight	Arms abducted to 90° with elbows straight	Arms abducted to 90° with elbows straight
**Bridge**	Arms by side	Clasp hands together, increase height of bridge	Clasp hands together, increase height of bridge	Clasp hands together, increase height of bridge

Participants were asked to attend three of four available one-hour classes per week for twelve weeks. Incentives for retention included a physical activity log, a yoga mat, light refreshments after each class, rewards for best attendance at the six week point (e.g., movie pass), a $100 stipend at follow-up, and a graduation certificate. The researchers monitored compliance by taking attendance at each class (ECB, ABK, EGW) and adherence was monitored and facilitated by the yoga class instructor.

### Primary Outcomes: Self-Report and Knee Strength

All primary outcome measures reflected the most symptomatic knee. Self-reported symptoms and physical function were evaluated using the Knee injury and Osteoarthritis Outcome Score (KOOS). The KOOS consists of 42 questions on a five-point Likert scale in five subscales: pain, other symptoms, activities of daily living, function in sport and recreation, and knee-related quality of life. Subscale scores were normalized out of 100, with 0 = extreme symptoms and 100 = no symptoms [52]. The KOOS produces highly reliable [53] and valid [54] data in knee OA.

Knee extensor and flexor strength were represented by the peak torques obtained during maximum voluntary isometric contractions (MVIC) of knee extension and flexion, expressed relative to body mass (Nm/kg). Participants were positioned on a dynamometer (Biodex Medical Systems, Inc., Shirley, NY, USA) such that the knee was in 65° of knee flexion and the knee joint centre was in line with the dynamometer axis. The ankle cuff (point of force application) was placed immediately proximal to the medial and lateral malleoli (average distance to dynamometer axis = 29.3±2.5 cm). Additional straps across the chest and pelvis minimized extraneous motions. After a submaximal isotonic warm-up and practice, participants completed five knee extensor MVICs and five knee flexor MVICs. Verbal encouragement was used to maximize effort during these contractions. The peak value from each of the five MVICs for extension and flexion efforts was extracted and normalized to body mass to represent strength.

### Secondary Outcomes: Mobility, Fitness, and Peak KAM

Mobility was captured using a Six-Minute Walk Test (6MWT), a 30-second chair stand test (30-s CST), and a stair-climbing protocol. The 6MWT measures the furthest distance a participant can walk in six minutes in an indoor, well-lit, rectangular hallway. The 6MWT was completed as per the published protocol and produces reliable data in knee OA [55]. The 30-s CST measured the number of times the participant could rise and sit back down from a standard chair in 30 seconds. The total number of completed stand-and-sit attempts before the trial ended was the score. This test produces reliable and valid data in people with knee OA [56]. Finally, the stair-climbing protocol assessed the time to ascend and descend (separately) a 9-step staircase as quickly and safely as possible, without running or skipping stairs. Recording time started when the participant’s lead foot left the ground and recording time stopped when both feet were planted firmly on the final step. A handrail was available. For each of stair ascending and descending, the mean of two attempts was recorded as a score [55]. In 30 healthy adults, these stair ascent and descent tasks produced reliable data (ICC 0.72–0.88, SEM <0.4s) in our laboratory.

Participants were asked to complete a cycle ergometer submaximal test to yield an estimated maximal oxygen consumption score (mL/kg/min). The YMCA cycle ergometer submaximal test estimates maximal oxygen consumption from submaximal data points [57]. This test is ideal for assessing the cardiovascular fitness of individuals not accustomed to maximal intensity exercise. The protocol included three or more consecutive 3-minute stages at a given workload. The target was to raise the participant’s heart rate (measured with a heart rate monitor) to a value between 110 beats per minute and approximately 85% of the age predicted maximum heart rate for two consecutive stages. The initial stage consisted of a 25 Watt workload at a pedaling rate of 50 revolutions per minute. The workload of subsequent stages was determined by heart rate during the final minute of the first stage. When the target heart rate was achieved for two consecutive stages, the test was terminated. Oxygen cost was estimated for the final two stages using a formula encompassing workload, body mass, and derived constants (S2 Appendix). The predicted values from submaximal tests have been cross-validated to the traditional gold standard methods of achieving a true maximal oxygen consumption (via maximal treadmill running) [57].

The KAM was calculated from motion and force data and expressed relative to body mass (Nm/kg) from the most symptomatic knee. The peak KAM during gait was captured. Each participant was instrumented with four clusters of infrared emitting diode (IRED) markers. These clusters were placed on the lateral foot, shank, and thigh segments of the involved limb and the sacrum. Three-dimensional motion of these clusters during gait and the yoga exercises was collected at 100 Hz with a 9-camera (3 banks) motion capture system (Optotrak Certus, Northern Digital Inc., Waterloo, ON). Synchronized with this kinematic collection, kinetic data were recorded using four floor-embedded force plates (OR6-7, Advanced Mechanical Technologies Inc, Watertown, MA) at 1000 Hz. For the gait analysis, each participant was instructed to walk down a 10-metre walkway barefoot at a self-selected pace. Trials were considered acceptable when the participant completed a full heel-strike and toe-off on a force plate with the involved limb. Five complete trials were collected for each participant.

### Tertiary Outcomes: Biomechanical Measures

All tertiary outcomes were captured from the limb of the most symptomatic knee. Kinematic and kinetic data were collected as described above. Analyses of four yoga postures (squat and lunge exercises) were completed barefoot. We selected four postures to ensure neuromuscular fatigue did not confound the measures. We selected these four postures because these formed the basic position of multiple postures used in the intervention that required variations on arm placement. The squats were “legs-together” (feet hip width apart) and “wide-legged” (feet externally rotated and shoulder-width apart). Both squats involved as much knee and hip flexion as possible while maintaining balance and comfort. Alignment instructions included the following: with the involved limb on one force plate, align knees over the 2^nd^ toes, maintain a neutral lumbar lordosis, and flex shoulders to 90° (elbows extended). The lunges involved a staggered stance. The aim was to flex the forward or “leading” knee to 90°. In the “trailing” leg, the knee was extended and the foot was externally rotated to 45° (as able) to maintain heel-floor contact. Alignment instructions were as follows: ensure heels align in the sagittal plane, position the leading knee over the 2^nd^ toe, maintain pelvic alignment in the frontal plane, and flex shoulders to 90° (elbows extended). Participants were asked to flex the leading knee as much as possible while maintaining balance and comfort. Lunges were repeated such that the involved limb was in each the leading and trailing position, with the involved limb placed on the force plate during both postures. For all squats and lunges, each position was held for ten seconds and repeated three times.

Using these kinematic and kinetic data, the KAM was calculated using inverse dynamics (C-Motion Inc., Kingston, ON) [58]. The joint coordinate system using a X-Y-Z Cardan sequence was used to calculate knee moments in Visual 3D [59]. The static portion of each squat and lunge trial (calculated within ± three standard deviations of the mean KAM from the middle of the trial) was selected and a moving average of the coefficient of variation was calculated. A three-second window within the static portion from the lowest coefficient of variation value was chosen for analysis. Mean KAM during this window was calculated and an average of two completed trials were computed (Mathworks Inc., Natick, MA).

Normalized mean muscle amplitude was captured using electromyography (EMG) during the yoga postures and presented as a percentage of MVIC (%MVIC). After preparing skin by shaving and wiping with isopropyl alcohol, silver-silver chloride dual electrodes (Natus Neurology Inc., Middleton, WI) were affixed to the skin over the biceps femoris, rectus femoris, semitendinosus, vastus lateralis, and vastus medialis muscles along the orientation of the muscle fibers (www.seniam.org, Enschede, Netherlands). Time-synchronized with force and motion data, raw EMG signals were bandpass filtered at 10–500 Hz and sampled at 1500 Hz (Desktop Telemyo DTS, Noraxon, Scottsdale, AZ). Each muscle was collected with a wire-minimal EMG sensor equipped with a reference electrode (dual differential amplifier, CMRR > 100 dB, input impedance > 100 MΩ).

For each trial, the mean amplitude of EMG signal during each exercise was calculated from the same analysis window selected for the KAM (Mathworks Inc., Natick, MA). All EMG data had bias removed, were full-wave rectified, and linear-enveloped using a 2^nd^ order low-pass zero lag Butterworth filter with a cut-off of 6 Hz (Mathworks, Inc., Natick, MA). Peak activations from MVICs were used to normalize EMG amplitude. EMG data represented the average of two completed trials for each static exercise.

### Sample Size Estimation

Sample size estimation for a t-test between two time points in one group was determined *a priori* for the primary outcomes of self-reported symptoms and knee strength. Systematic reviews of the effectiveness of resistance training for improving self-reported symptoms including pain and isometric knee extensor and flexor strength in knee OA had average effect sizes of -2.11 [9] and 0.41 [60] respectively. Given the lower effect size (0.41) and a Type I error = 0.05 on a 1-tailed test (based on strong evidence of improvement in symptoms [61] and strength [48]), a sample of 39 participants was chosen to yield 80% power to detect a significant effect. We aimed to recruit 45 participants to account for drop-outs and losses to follow-up.

### Statistical Analyses

Descriptive statistics were calculated. Normality of the outcomes was assessed using the Shapiro-Wilk test. Missing data were treated using case-wise deletion. Statistical analyses were completed in SPSS (IBM, Versions 21 and 22).

The primary analyses evaluated whether the yoga strengthening program improved self-reported outcomes (KOOS subscales) and knee strength (extensor, flexor torques) from baseline to follow-up using 1-tailed paired t-tests for normally distributed data. The secondary analyses evaluated whether the yoga strengthening program improved mobility performance (6MWT, 30-s CST, stair-climbing) and fitness, and decreased peak KAM during gait from baseline to follow-up using 2-tailed paired t-tests for normally distributed data. A 2-tailed paired t-test was used for a more conservative statistical approach as mobility performance, fitness, and peak KAM are less frequently reported in the literature. If data were not normally distributed, related-samples Wilcoxon signed rank test was used. Multiple comparisons were adjusted using a Bonferroni correction.

For the tertiary analyses, a one-way analysis of variance (ANOVA) and a Tukey HSD post hoc test (or for non-normal data, a Kruskal-Wallis one-way ANOVA) compared the average KAM during yoga postures with the peak KAM during gait. Also, the normalized mean EMG amplitudes for lower limb muscles were compared between baseline and follow-up using 2-tailed paired t-tests, or paired-samples Wilcoxon signed rank tests, as appropriate.

## Results

### Participants

Recruitment took place in May-June 2014 and follow-up data collection was completed in August 2014. A total of 79 people were assessed for eligibility. People were excluded for the following reasons: did not meet the inclusion criteria (n = 11), declined to participate (n = 18), and other (n = 5). Forty-five participants enrolled, 39 completed follow-up, and data from 38 participants were analyzed (Fig 1). Of the six participants that did not complete follow-up, one was an adverse event (undiagnosed ankle injury) unrelated to the intervention. Two participants developed disqualifying knee symptoms (patellofemoral pain), which may be a potential harm of the intervention. Two participants dropped out due to time commitment, and one participant dropped out due to a chronic respiratory illness. One participant completed the study but commenced wearing a knee brace during the intervention. Data from this participant were excluded. Table 2 outlines participant baseline characteristics for the 38 participants whose data were included in the analyses. The average number of classes attended per week was 2.6 (0.4). The average absolute number of classes attended was 31.3 (8.7), or an average adherence rate of 87.1%.

**Table 2:** Baseline characteristics of 38 women with symptomatic knee OA who completed the yoga intervention.

Variable	Mean (Standard Deviation)	[Minimum, Maximum]
Age (years)	60.3 (6.5)	[50, 77]
Body Mass Index (kg/m^2^)	29.5 (5.3)	[21.1, 43.9]
Height (m)	1.63 (0.06)	[1.50, 1.72]
Body mass (kg)	78.1 (14.8)	[51.2, 115.4]
Waist Circumference (cm)	93.9 (11.5)	[70, 123]
Classes Attended (/week)	2.6 (0.7)	[0.8, 3.6]

No statistical differences were observed for any demographic variables between those that did and did not complete follow-up (p>0.05). The drop-out group had a significantly lower KOOS Quality of Life score than the group that completed follow-up suggesting the drop-out group had a decreased knee-related quality of life at baseline. No other primary or secondary outcome measures were statistically different between the two groups. No tertiary outcome measures were statistically different between those that completed and those that did not complete follow-up (p>0.05).

All participants completed the fitness test; however we were unable to calculate a score for five participants at baseline and six participants at follow-up. Two factors precluded calculation. First, some participants were unable to maintain the required cadence. Second, based on heart rate responses, the test was terminated before two stages of the test were completed. Two stages were necessary to calculate a score. Finally, KAM and raw EMG data were analysed for 36 participants due to equipment issues with two participants (kinematic calibration issue).

### Primary Outcomes: Self-Report and Knee Strength

The Shapiro-Wilk test showed that data for all self-report outcome measures were normally distributed. Table 3 summarizes the outcome measures at baseline and follow-up for self-reported symptoms and physical function. Improvements were noted in all subscales of the KOOS (p<0.001). The pain, symptoms, activities of daily living, and quality of life subscales improved 16.3–20.3%. The greatest improvement occurred in the sports and recreation subscale, with a 42.5% improvement at follow-up compared to baseline.

**Table 3:** Mean (Standard Deviation) at baseline and follow-up for self-reported physical function (Knee injury and Osteoarthritis Outcome Score). Mean difference scores (follow-up–baseline) with 95% confidence intervals (CI) are also given, with positive change indicating improvement in self-reported outcomes and strength. Multiple comparisons for all outcome measures were corrected using a Bonferroni correction (new alpha level was set to p = 0.007). Significant comparisons are denoted with an asterisk (*). ADL–Activities of Daily Living

Self-Reported Outcomes [KOOS (/100)]	Baseline (Week 0) Mean (SD)	Follow-up (Week 12) Mean (SD)	Mean Difference [95% CI]	Significance
Pain	67.7 (15.4)	79.4 (12.7)	12.0 [7.9, 16.1]	<0.001*
ADL	74.9 (15.8)	87.1 (11.1)	11.3 [6.6, 16.1]	<0.001*
Symptom	63.5 (17.5)	74.6 (16.6)	12.5 [8.6, 16.4]	<0.001*
Sports and Recreation^ɸ^	48.9 (25.0)	69.6 (24.0)	20.1 [13.2, 27.0]	<0.001*
Quality of Life^ǂ^	49.0 (19.8)	59.0 (20.0)	101. [5.8, 14.3]	<0.001*

^ǂ^Missing due to incomplete questionnaire (n = 1)

^ɸ^Missing due to incomplete questionnaire (n = 3)

Table 4 summarizes the baseline and follow-up results for isometric strength. Knee extensor strength data were normally distributed. Knee extensor strength increased 5.6% from baseline to follow-up (1.8±0.6 to 1.9±0.6 Nm/kg; p = 0.004). A related-samples Wilcoxon signed rank test was used to examine change in knee flexor strength. Knee flexor strength increased from 0.7±0.3 Nm/kg to 0.8±0.3 Nm/kg from baseline to follow-up (14.3% increase; p = 0.001).

**Table 4:** Mean (Standard Deviation) at baseline and follow-up for strength. Mean difference scores (follow-up–baseline) with 95% confidence intervals (CI) are also given, with positive change indicating improvement in self-reported outcomes and strength. Multiple comparisons for all outcome measures were corrected using a Bonferroni correction (new alpha level was set to p = 0.007). Significant comparisons are denoted with an asterisk (*).

Strength (Nm/kg)	Baseline (Week 0) Mean (SD)	Follow-up (Week 12) Mean (SD)	Mean Difference [95% CI]	Significance
Knee Extensor Torque	1.8 (0.6)	1.9 (0.6)	0.1 [0.0, 0.2]	0.004*
Knee Flexor Torque	0.7 (0.3)	0.8 (0.3)	0.1 [0.0, 0.1]	0.001*

### Secondary Outcomes: Mobility, Fitness, and Peak KAM

The Shapiro-Wilk test demonstrated that of the mobility performance and fitness measures, only the 6MWT data were normally distributed. Table 5 summarizes the outcome measures at baseline and follow-up for mobility and fitness. Participants demonstrated a 7.1% improvement in 6MWT scores (535.2 to 573.0 m, p<0.001) and a 9.8% improvement in the 30-s CST (13.3 to 14.6 repetitions, p = 0.006). Stair ascent and descent time did not change between time points.

**Table 5:** Mean (Standard Deviation) at baseline and follow-up for mobility performance. Mean difference scores (follow-up—baseline) with 95% confidence intervals (CI) are also given, with positive change indicating improvement in mobility performance and fitness. Multiple comparisons for all outcome measures were corrected using a Bonferroni correction (new alpha level was set to p = 0.008). Significant comparisons are denoted with an asterisk (*).

Mobility Performance	Baseline (Week 0) Mean (SD)	Follow-up (Week 12) Mean (SD)	Mean Difference [95% CI]	Significance
Six-Minute Walk Test (m)	535.2 (99.1)	573.0 (102.6)	37.7 [21.9, 53.5]	<0.001*
30-second Chair Stand Test	13.3 (3.7)	14.6 (4.9)	1.3 [0.4, 2.2]	0.006*
Stair Ascent	5.26 (2.86)	4.90 (2.02)	-0.36 [-0.78, -0.06]	0.124
Stair Descent	4.64 (2.69)	4.63 (3.92)	-0.01 [-0.54, 0.52]	0.171

Table 6 shows results of fitness and peak KAM. The estimated maximal oxygen consumption was the same at baseline and follow-up (p = 0.372) for the subsample (n = 32) for which estimated values were calculated. The peak KAM during gait was unchanged at follow-up (p = 0.524).

**Table 6:** Mean (Standard Deviation) at baseline and follow-up for fitness and peak KAM during gait. Mean difference scores (follow-up—baseline) with 95% confidence intervals (CI) are also given, with positive change indicating improvement in mobility performance and fitness. Multiple comparisons for all outcome measures were corrected using a Bonferroni correction (new alpha level was set to p = 0.008). Significant comparisons are denoted with an asterisk (*).

Fitness and Peak KAM	Baseline (Week 0) Mean (SD)	Follow-up (Week 12) Mean (SD)	Mean Difference [95% CI]	Significance
Estimated Maximal Oxygen Consumption (mL/kg/min)^˩^	26.3 (5.4)	26.8 (5.1)	0.5 [-0.6, 1.7]	0.372
Peak KAM	0.42 (0.16)	0.43 (0.15)	0.01 [-0.02, 0.04]	0.524

^˩^Participants completed the protocol, but, in some cases, their cadence or heart rate responses precluded calculation of estimated values at baseline (n = 5) and follow-up (n = 6). No fitness data from these participants are included in the table.

### Tertiary Outcomes: Biomechanical Measures

Baseline KAM data were not normally distributed. Average KAM during the four yoga postures are presented in Table 7 and were nominal at both baseline and follow-up. Results from the Kruskal-Wallis one-way ANOVA revealed that average KAM values during all four exercises were lower than that of peak KAM during gait at baseline (p<0.001).

**Table 7:** Knee adduction moment (KAM) normalized to body mass during four yoga postures to strengthen knee muscles (Nm/kg). Biomechanical data are presented on n = 36 participants.

Yoga Posture	Baseline (Week 0) Mean (SD) [Min, Max]	Follow-up (Week 12) Mean (SD) [Min, Max]
Legs-together Squat	-0.04 (0.14) [-0.48, 0.34]	-0.07 (0.12) [-0.31, 0.20]
Wide-legged Squat	-0.02 (0.14) [-0.36, 0.28]	-0.03 (0.11) [-0.28, 0.25]
Lunge (Leading Leg)	0.08 (0.13) [-0.22, 0.38]	0.05 (0.10) [-0.25, 0.25]
Lunge (Trailing Leg)	-0.07 (0.14) [-0.29, 0.33]	-0.12 (0.12) [-0.53, 0.23]

Data were not normally distributed for the hamstrings normalized mean EMG amplitudes across yoga postures. Vastus medialis normalized mean EMG amplitude was not normally distributed for the lunge postures. Rectus femoris normalized mean EMG amplitude was not normally distributed for the leading leg of a lunge only. Normalized mean EMG amplitudes ranged from 7.3 to 31.0% at both baseline and follow up. Following an adjustment for multiple comparisons, normalized mean EMG amplitude in the five lower limb muscles did not change from baseline to follow-up for all four postures.

## Discussion

We explored whether a novel strengthening exercise program for knee OA, designed specifically to minimize the KAM, would be useful to pursue. Exposure to large KAM has been implicated in the structural progression of this disease [26–28,62]. It is important to ensure that treatment options for knee OA promote physical activity but do not incorporate exposures to this risk factor for worsening the disease. The proposed yoga-based strengthening program aims to enable people with knee OA to exercise without contributing a cumulative exposure to large KAM. After completing this yoga strengthening program in this sample of women with knee OA, we observed improvements in all subscales of the KOOS (pain, symptoms, activities of daily living, sports and recreation, and quality of live) and knee extensor and flexor strength compared to baseline. Further, two of three mobility performance measures improved at follow-up compared to baseline; however fitness and peak KAM during gait were unchanged. Consistent with our tertiary hypothesis, the average KAM during the yoga postures was lower than the peak KAM experienced during gait. However, inconsistent with our initial hypothesis, the mean normalized EMG amplitudes during yoga postures were also unchanged between baseline and follow-up. Normalized mean EMG amplitudes during the strengthening exercises ranged between 7.3 and 31.0% of maximum effort contractions. These data suggest this magnitude of stimulus related to improved strength. These data show promise that an innovative “low KAM” yoga strengthening program could improve self-reported outcomes, knee strength, and mobility performance in women with knee OA.

The ideal type of strengthening for knee OA is unknown [17]. Strengthening intervention studies have implemented a variety of exercises. Examples include simple quadriceps exercises (e.g., straight leg raises); non-weight bearing exercises using cuff-weights, Therabands, and dynamometers; weight-bearing exercises; and neuromuscular programs to enhance sensorimotor function [6,14–16]. Many chose non-weight-bearing exercises to minimize the knee compressive load, though these exercises have little functional relevance [17]. The exercise program proposed in the current study marks the first attempt that we are aware of to design a strengthening program with functional relevance based on the biomechanical loads.

While we cannot comment on the efficacy of the program without a RCT design, it is unlikely that improvements across several domains, including self-reported symptoms and physical function, strength, and mobility were spontaneous. Clinical improvements observed in the current sample are equivalent to improvements reported after traditional exercise programs. The magnitude of improvement in KOOS, strength, and mobility were similar to that found in other studies [14,15,38,63]. For example, an 18-month RCT of 454 participants with knee OA compared three groups receiving diet, exercise, or both. The exercise-only group demonstrated 28% and 24% improvements on pain and function subscales of the Western Ontario and McMaster Universities Osteoarthritis Index respectfully [63]. Despite our smaller sample size and shorter intervention, findings from the current proof-of-principle study demonstrated similar improvements. Further, we observed improvements greater than minimal detectable change (MDC) data for the KOOS symptoms and sports and recreation subscales [64]. We were unable to identify MDC or minimal clinically important difference (MCID) values for mobility performance or strength outcomes based on exercise interventions in knee OA. Improvements observed for the 6MWT [55] and strength [65] were approximately half of the estimated MDC determined from data before and after arthroplasty. While these improvements in strength appear relatively small and the clinical relevance of these strength increases are unclear, the 5.6–14.3% increase in strength values found in the current study are within the range expected in strengthening programs in knee OA [46]. A need exists for MDC and MCID data relevant to exercise. Nonetheless, clinical improvements in symptoms, strength and mobility performance following this novel program for knee OA must be compared to change observed in a control group, using a RCT design, to establish efficacy.

While mobility performance was improved during the walking and sit-to-stand tasks, no improvement in stair-climbing was noted after participants completed this intervention. It is possible that this negative finding reflects training specificity. While participants completed yoga postures such as squats that likely translate into improvements in sit-to-stand activities, the intervention did not incorporate exercises that reflected stair-climbing. It is important to note, at baseline, that the participants ascended and descended nine stairs, on average, between 4s and 5s. These scores leave little room for improvement. A ceiling effect is likely responsible for the lack of change noted before and after the intervention [56]. The stair-climbing task may produce greater variability in a sample with greater mobility limitations, such as pre-surgical candidates.

Inconsistent with our hypothesis, the fitness test selected for this study demonstrated no differences between baseline and follow-up. It is not surprising that an exercise program tailored primarily to strengthen the quadriceps muscles was not a stimulus for improvement in fitness; though previous work has documented strength training can improve fitness in older adults [12]. It is important to note that the submaximal cycle ergometer test is likely not appropriate for people with knee OA. We initially chose this protocol to eliminate the influence of pain during weight-bearing; for example on a treadmill test. However, the cycle ergometry test used here required a pedaling cadence some participants could not complete. Also, the criteria for heart rate to terminate the test resulted in the inability to estimate a maximal oxygen consumption value for several participants. Thus, it is difficult to form conclusions about fitness changes between baseline and follow-up in this study.

We propose here that the KAM may be useful in setting guidelines for exercises for knee OA. While we initially hypothesized that the strengthening intervention would decrease peak KAM during gait, several studies show that exercise has no impact on the KAM during gait [37,38,66–69]. For example, a RCT of lower limb strengthening in 89 participants with knee OA compared the peak KAM during gait, self-reported pain and function, stair-climbing performance, and maximum isometric strength before and after 12 weeks of either a strengthening program or no intervention [37]. While these previous findings might initially appear discouraging, it is possible that, rather than attempt to change the KAM, we could use the KAM to tailor exercise. Due to high KAM values identified in previous work [44], the strengthening program used in the current study did not include single leg stance exercises shown to improve hip strength [14,67] and balance [70]. Indeed, in the current sample of women with symptomatic knee OA, analyses of the basic yoga postures (specifically squats and lunges, upon which variations of arm positions were used) revealed these exercises produced nominal mean KAMs. Thus, utilizing these exercises can promote physical activity among women with symptomatic knee OA without concerns of increasing cumulative exposures to large KAMs, which has been implicated in worsening structural knee OA.

Inconsistent with our initial hypothesis, we did not observe a reduction in normalized mean EMG amplitudes during the yoga postures between baseline and follow-up. We anticipated that increased torque output from knee extensors and flexors as a result of the strengthening program would result in concomitant decreases in normalized mean EMG amplitudes to complete the same exercise task. During the strengthening exercises, lower limb normalized mean EMG amplitudes ranged between 10.1–28.4% MVIC at baseline and 7.3–31.0% MVIC at follow-up. We observed relatively shallow knee flexion during the squats and lunges in this sample (e.g., 9.7–46.6 degrees at baseline and 7.1–50.8 degrees at follow-up) which may account for the low muscle amplitudes. Future work should explore whether increases in peak torque were due to neural changes versus hypertrophy of muscle fibre tissue.

The intervention appeared to be well-accepted by the study participants. Mean weekly attendance was 2.6±0.7 classes and adherence was 87.1% across the 12-week intervention period. All but one participant attended at least one class per week. Our target was 2.5 classes per week because clinical outcomes in knee OA improve after one supervised class per week; however the effect size increases with greater number of contacts [6].

This study had limitations. Foremost, because this study did not utilize a RCT design, it is unclear whether the yoga strengthening program was responsible for clinical improvements observed between baseline and follow-up, or another confounding variable. For example, the positive findings may be a result of attention bias. We engaged in this simple, relatively low cost design to establish proof-of-principle. Future work utilizing a RCT design is necessary. Second, the KAM represents the net reaction moment that does not account for muscle contributions and thus may underestimate internal joint loads. Therefore, while the KAM may be low during the yoga exercises presented here, other contributors to joint load such as agonist-antagonist muscle co-activity may contribute to substantial load on the medial knee compartment. Nonetheless, KAM was related to joint contact forces directly measured in the knee (R^2^ = 0.77, p<0.001) [71]. Since an increase in KAM has been related to disease progression [26], designing an exercise program that incorporates low KAM exercises may aid in disease management while improving self-reported outcomes, strength, and mobility. Third, the program is likely inappropriate for individuals who experience patellofemoral pain symptoms. While the proposed strengthening program minimizes KAM, it demands large patellofemoral loads. Fourth, the YMCA submaximal cycle ergometer test was not appropriate for this sample. Fifth, the findings may not be generalizable to men with knee OA. Finally, participants in this study may not have radiographic disease, or disease confined to the medial compartment. Ultimately the goal of the OA-specific strengthening program is to improve clinical outcomes, suggesting an emphasis on symptomatic disease is required.

This study showed that a strengthening program designed to minimize exposure to large magnitude KAMs had promise in improving self-reported symptoms and physical function, and strength among women with clinical knee OA. Two of the three mobility performance tests (walking and sit-to-stand) improved at follow-up compared to baseline, however fitness and peak KAM during gait did not change following the intervention. Unfortunately, the assessment of fitness using the YMCA submaximal cycle ergometry protocol resulted in missing data due to incomplete tests. Also, the stair-climbing measurement used likely yielded a ceiling effect in this clinical sample. Finally, it is important to note that the average KAMs during the yoga postures were lower than that during normal gait, providing confidence that these yoga postures may be useful as a treatment strategy for knee OA. However, strengthening the knee extensors and flexors did not lower muscle activation amplitudes as a result of this strengthening program. It seems reasonable that an exercise program designed with low KAM exercises may aid in disease management while improving self-reported outcomes, strength, and mobility. Further investigation using an RCT design is needed to make conclusions regarding the efficacy of this OA-specific strengthening program in people with knee OA.

## Supporting Information

S1 Checklist The TIDieR (Template for Intervention Description and Replication) Checklist. (PDF)

S1 Protocol Submitted ethics protocol. (DOCX)

S1 Appendix Biomechanics exercise program with instructions and progressions. (DOCX)

S2 Appendix YMCA submaximal VO2 cycle ergometry test protocol and data collection package. (DOCX)
