# Supplementary material for: A Yoga Strengthening Program Designed to Minimize the Knee Adduction Moment for Women with Knee Osteoarthritis: A Proof-Of-Principle Cohort Study
Source: PLoS One. 2015 Sep 14;10(9):e0136854. doi: 10.1371/journal.pone.0136854 (PMC4569287; doi:10.1371/journal.pone.0136854)
Supplement: S1 Protocol — (DOCX) [file pone.0136854.s002.docx]

**A Yoga Exercise Intervention Designed for Women with Knee Osteoarthritis**

**Protocol**

**Introduction**

Over 4 million Canadians endure a form of arthritis (1). This number will escalate because, as a nation, we are getting older and heavier at an alarming rate (1). Arthritis is the single greatest cause of chronic disability among community-dwelling older adults, by painfully restricting mobility in daily life (2). The most common arthritis, osteoarthritis (OA), affects 10% of Canadian adults (1). Osteoarthritis results from excessive and abnormal loads that degrade cartilage and bone, most frequently in the knee. Adding to the complexity of treatment, knee OA rarely occurs in isolation. This population has elevated incidences of cardiovascular and gastrointestinal disorders and depression (1).

Keeping older adults with knee OA moving is critically important but not straight-forward. Systematic reviews and clinical practice guidelines confirm that exercise is critical in knee OA treatment (3-5). Exercise reduces symptoms, co-morbidity and improves physical function (3-5). In fact, the effects of aerobic and strengthening exercise on pain are equivalent to medication (5). However, important caveats exist. Generic exercise prescription in large samples produces mediocre findings in terms of pain and physical function (3). It is important to note that these exercise programs did not benefit from data that would properly dose the type, intensity, repetition and biomechanical load of the exercise for people with knee OA.

There are concerns that improperly dosed exercise can accelerate knee OA progression. High loading repetition, a feature of walking programs for example, is linked to greater intensity knee pain in people with knee OA (6). Exercise programs that require footwear may not be ideal because shoes elevate the mechanical loads within the knee. In particular, the knee adduction moment was increased by 38% when 68 subjects were wearing running shoes compared to barefoot (7). The knee adduction moment reflects the proportion of load placed on the medial knee compartment that is most commonly degraded in this disease. Longitudinal studies clearly demonstrate that the knee adduction moment predicts progression noted on radiographs (8) and on magnetic resonance imaging scans (9). Finally, while quadriceps strengthening improved symptoms in many samples with knee OA, concerns have been raised about the potential for traditional strengthening programs to increase the contact forces that may contribute to acceleration of knee OA (10, 11). These experts recommend that strengthening exercises must elicit muscle activation patterns, while maintaining ideal alignment of the lower extremity to minimize the knee adduction moment.

Studies showing that generic exercise programs improved pain and mobility in people with knee OA provide confidence that exercise is fundamental to the management of this disease. Improving exercise programs to minimize biomechanical risks specific to knee OA may enhance the benefits of exercise for this population. Yoga offers a foundation of static postures that improve muscle strength and joint flexibility, while eliminating repetition, footwear and, if properly analyzed, the knee adduction moment.

Yoga is an exercise activity that emphasizes controlled, fluid movements through a variety of static postures. Many yoga postures challenge and strengthen musculature of the lower extremity, while minimizing some potential negative effects that other exercise programs pose for people with knee OA. For example, running is an activity that includes large and repetitive loads on the knee, which may not be the best-suited activity for individuals with knee OA. Yoga has beneficial effects on various aspects of quality of life, including improved cardiorespiratory health, body awareness, well-being and mental health. Further, yoga has been shown to improved strength, balance, flexibility, pain, and disability in older adults and individuals with knee OA (12) (13) (14) (15) (16) (17). For example, a pilot study consisting of 7 women over age 50 years with symptomatic knee OA implemented an 8-week program of modified yoga postures. Each week participants attended one 90-minute weekly class. Participants reported improvements in knee pain, physical function and mental health, demonstrating that yoga holds great potential as a treatment option for older, yoga-naive women with knee OA (16).

We recently completed a biomechanical analysis of ten common yoga postures that emphasize lower extremity strengthening, including squats, lunges and balancing postures in 30 young women [not yet published]. These analyses focused on the knee adduction moment and muscle activation patterns to identify postures that did not overload the medial knee while requiring enough muscle activity to result in strengthening. Of the ten yoga postures, eight produced negligible knee adduction moments and all required muscle activations that would facilitate strengthening of the quadriceps and hamstrings [not yet published]. These data provide the foundation for an intervention that emphasizes the eight yoga postures that result in negligible knee adduction moments.

Research Question & Hypotheses

The primary objective of this study is to determine whether a yoga exercise intervention will improve the strength of the musculature that surrounds the knee without increasing knee pain in women with knee osteoarthritis (OA). A secondary objective is to determine whether a yoga exercise intervention will reduce the knee adduction moment during gait, improve mobility performance, and improve cardiovascular fitness.

Study participants will experience increased strength in the musculature surrounding the knee and reduced knee pain after completing the yoga intervention. As well, study participants will demonstrate a reduced knee adduction moment during gait, improved mobility performance, and improved cardiovascular fitness after completing the yoga intervention.

**Study Design and Methods**

This intervention study features a repeated measures design. Outcome measures will be assessed at McMaster University before and after a 12 week intervention. The yoga exercise intervention will be delivered at a yoga studio in downtown Hamilton, Ontario. This proof of principle study will aim to provide preliminary data supporting the use of a yoga exercise intervention in a sample of women with knee OA.

Participants

Fifty community-dwelling women, 50 years of age and older, who meet the American College of Rheumatology (ACR) criteria for clinical knee OA will be recruited to participate in this study. The ACR clinical criteria require 3 of the following:

- Knee pain on most days of the week,
- Less than 30 minutes of morning stiffness,
- Crepitus with active range of motion,
- Bony enlargement,
- Bony tenderness to palpation, and
- Signs of inflammation (warmth, swelling).

*Inclusion and Exclusion Criteria*

Women were selected because incidence of knee OA is two times greater among women than men over the age of 50 years (18-20). Muscle weakness is of particular concern in women with knee OA because their absolute muscle strength is lower compared to men, and therefore women are closer to the critical thresholds of strength required for activities of daily living (21). Finally, women report pain secondary to knee OA more frequently than men (20) perhaps because the pain experience is different (22). The incidence and prevalence of knee OA increases with age, particularly in women (18, 20, 23-25).

The exclusion criteria for participation include a self-reported history of patellofemoral symptoms. The exercise intervention is focused on unloading the medial knee compartment; however these exercises could place large loads on the patellofemoral joint, such as squats and lunges. In addition, participants that self-report osteoporosis will be excluded. The exercise intervention includes spinal flexion and therefore may increase the risk of a fragility fracture in the spine. Exclusion criteria include diagnosis of other forms of arthritis (e.g., rheumatoid, psoriatic); active non-arthritic knee disease (e.g., gout); conditions that might be exacerbated by the protocol (e.g., unstable angina); neurological conditions such as a stroke; and current/past use of intra-articular therapies or knee surgeries and; contraindications to magnetic resonance imaging (e.g., pacemaker). Participants will be excluded if they have a skin allergy to medical tape, use an adaptive aid such as a cane or cannot climb 2 flights of stairs safely. Lower extremity trauma within 3 months, ipsilateral hip or ankle conditions, radiation (e.g., cancer treatment) and pregnancy will also be exclusion criteria. Finally, participants that have not completed an annual physical examination by a physician in the past year will be excluded. We require this exclusion criterion to minimize the potential that participants have an underlying health condition that has not been identified or managed.

*Recruitment*

Two methods of recruitment will be used. First, participants will be recruited through a list of potential participants provided by Dr. Rick Adachi at the Centre for Appendicular Magnetic Resonance Imaging (CAMRIS) at St. Joseph’s Hospital. All of the individuals on this list have signed a consent form to be contacted for research studies. These potential participants will be identified by a receptionist at CAMRIS and a letter will be sent by the receptionist on behalf of CAMRIS inviting their participation. The letter will invite participants to email or call a research assistant if interested in the study. A research assistant will respond to potential participants using the same mode of communication. Once in contact, the research assistant will describe the purpose, protocol, risks and benefits of the study. Potential participants who express an interest will be screened for the inclusion and exclusion criteria.

Second, posters will be displayed in the Hamilton, Ontario community, including at the yoga studio. As well, the poster will be included in weekly electronic newsletters sent out by the yoga studio. The advertisement will invite potential participants to contact a research assistant via telephone or email. A research assistant will respond to potential participants using the same mode of communication. Once in contact, the research assistant will describe the purpose, protocol, risks and benefits of the study. Potential participants who express an interest will be screened for the inclusion and exclusion criteria. Dr. Rick Adachi will first meet with the potential participant at CAMRIS to confirm the presence of clinical knee OA.

For both recruitment strategies, any potential participant that expresses interest and meets the criteria for the study will be sent a copy of the consent form (electronic or hard copy at the potential participant’s discretion) and a map directing them to pre-paid parking. An appointment will be set at the MacMobilize Laboratory, McMaster University. At this time, participants will be instructed to avoid any high intensity physical activity for 24 hours prior to testing.

Yoga Intervention

The exercise intervention will be a 12-week yoga program requesting attendance at 3 supervised classes each week. Over each week, 4 classes will be available (Sunday 10-11am ; Tues, Thurs, Fri 3-4pm). Two 12-week sessions will be provided to ensure no more than 25 participants are in each session. We aim to start session one in November 2013 and session two in February 2014.

This class will be conducted at a yoga studio near downtown Hamilton in a regular temperature room (De La Sol Yoga located at the corner of York and Locke Streets; http://www.delasolyoga.com). The class will be instructed y Andre Grandbois. He is a certified yoga instructor (training completed in 2008). The yoga studio is a commercial space with dedicated free parking, yoga mats and other equipment, bathroom facilities including showers, and an elevator. A structured fee is provided to the instructor and yoga studio to access space, parking and equipment. The participants will incur no costs associated with attending the yoga classes.

Each class will be 1 hour in length. The yoga class will include a warm-up period featuring large body movements with no resistance. Then the instructor will lead the class through a series of standing postures identified with our previous work as requiring high muscle activity from the knee musculature, but minimal knee adduction moment. Examples of these postures include squats, lunges, wide-legged stances in various ranges of knee motion, and balance poses. The instructor will offer a variety of modifications for each pose, to ensure that the participants have an alternative best suited for their experience level. In addition, a series of seated poses that focus on flexibility will be completed. Each class will conclude after a guided deep-breathing and meditation activity. Throughout the class, education about knee joint anatomy, osteoarthritis and proper lower extremity alignment will be included.

Retention strategies to enhance adherence to the exercise intervention will include the following:

- Exercise logbook to track activity,
- $100 stipend upon completion of the final data collection,
- Draw for $5 Tim Horton’s gift certificate at the conclusion of some classes, and
- Rewards for best attendance across 12-weeks including a 3 month yoga pass, yoga mats, water bottles.

Outcome Measures

Outcome measures will be collected at baseline and after completion of the 12-week yoga intervention. These outcome measures will be collected at the MacMobilize Laboratory (Communications Research Laboratory Building, room B110) on McMaster campus. A study limb will be chosen as the most symptomatic knee.

*Primary Outcome Measures*

*A. Strength*

The peak torque developed during knee extension and flexion during a maximum voluntary isometric contraction will be measured by use of a Biodex System 2 isokinetic dynamometer. Data will be presented as Nm/kg. After a submaximal practice, 5 maximum-effort trials of isometric knee extension, with the knee joint positioned at 65°, will be recorded. The same protocol will be repeated for isometric knee flexion. The peak value for each of knee extension and flexion will be extracted. The reliability for these data are excellent (r=0.96) (26).

*B. Pain*

- The Knee Osteoarthritis Outcome Score (KOOS) is a patient-administered, 42 item questionnaire.  Study participants are asked to answer questions on a 5-point Likert scale, which takes approximately 10 minutes to complete.  The KOOS consists of 5 subscales:  pain, other symptoms, activities of daily living (ADL), function in sport and recreation and knee-related quality of life (QOL).  The questionnaire results in a normalized score out of 100 for each subscale, where 100 indicates no symptoms and 0 indicates extreme symptoms. The questionnaire relies on subject’s recall from the previous week.

The KOOS has been used in persons 13-79 years of age. The KOOS reference values are available from various sample populations including; age/sex based samples (33) (34), active soccer players (35), and male and female patients post anterior cruciate ligament reconstruction (36). The KOOS has a high test-retest reliability demonstrated by intra-class correlation coefficients of 0.80-0.97 for the Pain subscale, 0.74-0.94 for the Symptom subscale, 0.84-0.94 for the ADL subscale, 0.65-0.92 for the Sport/Rec subscale and 0.60-0.91 for the QOL subscale in patients with knee osteoarthritis (OA) (37). The minimal detectable change values for patients with knee OA are 13.4 for Pain, 15.5 for Symptoms, 15.4 for ADL, 19.6 for Sport/Rec, and 21.1 for QOL (38). Currently, the minimal clinically important change value is suggested to be 8-10 for the KOOS, proving the scale is sensitive to change (38). The KOOS has been reported to detect change following surgical procedures including; ACL reconstruction, meniscectomy, cartilage repair procedures, tibial osteotomy, total knee replacement and non-surgical procedures including; physical therapy, as well as nutritional and pharmaceutical interventions (39). The KOOS presents an internal consistency range (Cronbach’s α) of 0.65–0.94 for Pain, 0.56–0.83 for Symptoms, 0.78–0.97 for ADL, 0.84–0.98 for Sport/Rec and 0.71–0.85 for QOL in patients with knee OA (38). The KOOS has demonstrated convergent and divergent construct validity when compared against numerous instruments including the different subscales of the Short Form-36 and the Lysholm knee scoring scale (38).

The KOOS includes the complete and original WOMAC OA Index LK 3.0 therefore WOMAC scores can be calculated in isolation. The KOOS subscales ‘Sport and Recreation function’ and ‘Quality of Life’ have been shown to be more sensitive and distinct than the WOMAC subscales ‘Pain’, ‘Stiffness’, and ‘Function’ when studied in subjects with radiographic evidence of OA who underwent a meniscectomy 21 years prior to the study (mean age = 57 years, range = 38-76) compared to age and gender matched controls (40).

*Secondary Outcome Measures*

*A. Biomechanical Analyses of Gait and 3 Yoga Postures*

Participants will change into shorts, a sports bra, and be barefoot. The participants’ height, body mass, lower limb length, anterior superior iliac spine (ASIS) distance, posterior superior iliac spine (PSIS) distance, upper thigh circumference, abdominal circumference knee width, calf circumference and ankle width will be measured. Then, 3 rigid body marker clusters will be placed on the participant with Velcro straps. These will be placed between the left and right PSIS, on the study thigh, shank and the top of the foot. These correspond to the specifications required to create a lower-body model. Landmarks will be digitized in reference to the rigid body marker clusters. These landmarks include: both left and right ASIS, iliac crest, PSIS, greater trochanter, lateral and medial distal femoral condyles, lateral and medial tibial proximal condyles, lateral and medial malleoli, tibial tuberosity, proximal fibular head, head of metatarsals 1, 2, 5, and calcaneus.

With the participants on a force plate and in the field of view of three banks of Optotrak cameras (which record only positions of the markers), a 5 second standing reference trial will be collected for the motion capture system. Participants will be instructed to perform 10 hip rotations, 5 in each direction. Participants will be instructed to perform 5 knee flexions and extensions of both legs. Participants will be asked to perform 3 yoga postures (squat, lunge and wide-legged stance) for 10 seconds with at least a 10 second rest in between each posture. The order of these postures will be randomized between participants. Finally, participants will be asked to walk across the 10 foot motion capture area. For each yoga posture and gait, 3 successful trials will be recorded.

*B. Mobility Performance Measures*

- The Six-Minute Walk Test (6MW) is used to quantify walking ability. The 6MW is an inexpensive clinical tool that involves recording the distance that participants cover while walking indoors at their own pace for 6 minutes. Participants are free to stop or use a mobility aid to complete the walking task, making this measure clinically useful. The 6MW measurement will be recorded indoors in a well-lit, tiled rectangular hallway. The score recorded is the total distance traveled in 6 minutes. Instructions for the 6MW will correspond with the published protocol (44). The 6MW yields highly reliable (intraclass correlation coefficient=.96) and valid data (45).
- Participants will be asked to ascend and descend a 9-step staircase as quickly as possible, without compromising their safety. The hand rail can be used as needed. The participants will be asked to start at the base of the stairs at a distance from the first step of their choosing. The participants will be instructed not to run or jog, and not to skip any steps. Time to ascend and descend will be recorded separately, to the nearest 10^th^ of a second, using a stopwatch. Participants will be asked to repeat this procedure two times, where scores from only the ascent and descent on the second attempt are recorded. Data from our laboratory demonstrate excellent test-retest reliability of the stair ascent and descent tasks among 29 adults (ICC=0.881, 0.843 respectively).
- The 30 second chair stand task quantifies the number of sit-to-stand movements that can be completed within 30 seconds, starting from a seated position in an armless chair of a standard height (45 cm). After an opportunity to practice the task, participants will be asked to perform this task at a comfortable pace. Knee extensor strength has been shown to be a strong predictor of performance on repeated chair stand tests (46). In addition, lower extremity muscle power was predicted from performance of the 30 second chair stand in 14 older adults (47).

*C. Cardiovascular Measures of Fitness*

- In addition, participants will be asked to complete the YMCA cycle ergometer submaximal test. Maximal oxygen uptake (VO_2_max) is regarded as the best measure of cardiovascular fitness and is a good indicator heath status (48). Unfortunately, evaluating VO_2_max is expensive, time consuming, and requires specialized laboratory equipment, trained personnel, and medical supervision for some populations. The YMCA cycle ergometer submaximal test is used to estimate VO_2_max by extrapolating submaximal data points. This test is ideal for assessing the cardiovascular fitness of individuals not accustomed to maximal intensity exercise.

The protocol includes three or more consecutive 3-minute stages at a given workload that is designed to raise the participant’s heart rate to a value between 110 beats per minute and approximately 85% of the age predicted max heart rate for two consecutive stages. The initial stage consists of a 25 Watt workload at a pedaling rate of 50 revolutions per minute, which is maintained throughout the test. The workload of subsequent stages is determined by the participant’s heart rate during the final minute of the first stage. When a heart rate between 110 beats per minute and 85% of the age predicted heart rate max is achieved at a steady state for two consecutive stages the test is terminated. Oxygen cost is estimated for the final two stages using a formula encompassing workload, body mass and derived constants. The two oxygen costs are then used to formulate a linear equation, which is extrapolated to predict the participant’s VO_2_max. The submaximal test is used by the Canadian Society for Exercise Physiology and included in the Canadian Physical Activity, Fitness and Lifestyle Approach protocol. The predicted VO_2_max values from submaximal tests have been cross-validated to the traditional gold standard methods of achieving a true VO_2_max (via maximal treadmill running). A study examining 55 men (mean age = 38.1 ± 10.7) and 47 women (mean age = 38.7 ± 9.9) found a moderate correlation (r = 0.77, p ≤ 0.05) between the predicted VO_2_max values attained from the YMCA protocol and the true VO_2_max values from a treadmill test (49). This correlation is strengthened (r = 0.90, p ≤ 0.05) when the men are removed from the calculation, suggesting the relationship may be stronger in women, making the test more appropriate for the female sample population of the proposed study (49).

Protocol

*Baseline Measures: MacMobilize Laboratory at McMaster University, Hamilton*

1. Written, informed consent
2. Anthropometric Measurements
3. Resting Heart Rate and Blood Pressure
4. Instrumentation & Biomechanical Analyses (Gait and Yoga)
5. Mobility Performance Measures
6. Strength Assessment
7. Questionnaire: KOOS
8. YMCA protocol

This laboratory visit will require 2.5 hours to complete.

*Yoga Intervention: De La Sol Yoga at York and Locke Streets, Hamilton*

- 12-week intervention
- Requesting attendance to 3 supervised classes each week

Each yoga class will be 1 hour.

*Follow-up Measures*

1. Anthropometric Measurements
2. Resting Heart Rate and Blood Pressure
3. Instrumentation & Biomechanical Analyses (Gait and Yoga)
4. Mobility Performance Measures
5. Strength Assessment
6. Questionnaire: KOOS
7. YMCA protocol
8. Stipend

This laboratory visit will require 2.5 hours to complete.

Sample Size Estimation

Systematic reviews of the effectiveness of resistance training for improving self-reported physical function and isometric knee strength in knee OA had average effect sizes of 2.11 and 0.41 respectively (5). Given the lower effect size (0.41) and a Type I error = 0.05 on a 1-tailed test (based on strong evidence of improvement), a sample of 39 participants is necessary to yield 80% power to detect a significant effect. We aim to recruit 45 participants to account for drop-outs and losses to follow-up.

Statistical Analyses

Descriptive statistics will be determined for all of the outcome measures. Repeated measures analysis of variance will be used to determine if changes in the primary and secondary outcome measures as a result of the yoga exercise intervention. The p value will be set at p<0.05.

**References**

1. Badley E, Glazier R. Arthritis and related conditions: ICES Research Atlas. Toronto: Institute for Clinical Evaluative Sciences; 2004.

2. Guccione A. Arthritis and the process of disablement. Phys Ther. 1994;74:408-14.

3. vanBaar M, Dekker J, Oostendorp R, Bijl D, Voorn T, Bijlsma J. Effectiveness of exercise in patients with osteoarthritis of hip or knee: Nine month's follow-up. Ann Rheum Dis. 2001;60:1123-30.

4. Zhang W, Moskowitz R, Nuki G, Abramson S, Altman R, Arden N, et al. OARSI recommendations for the management of hip and knee osteoarthritis, part 1: Critical appraisal of existing treatment guidelines and systematic review of current research evidence. Osteoarthritis Cart. 2007;15:981-1000.

5. Fransen M, McConnell S. Exercise for osteoarthritis of the knee. Cochrane Database of Systematic Reviews. 2009;4.

6. Robbins S, Birmingham T, Callaghan J, Jones G, Chesworth B, Maly M. Association of pain with frequency and magnitude of knee loading in knee osteoarthritis. Arthritis Care Res. 2011;63:991-7.

7. Kerrigan D, Franz J, Keenan G, Dicharry J, Della Croce U, Wilder R. The effects of running shoes on lower extremity joint torques. PMR. 2009;1:1058-63.

8. Miyazaki T, Wada M, Kawahara H, Sato M, Baba H, Shimada S. Dynamic load at baseline can predict radiographic disease progression in medial compartment knee osteoarthritis. Ann Rheum Dis. 2002;61:617-22.

9. Bennell K, Bowles K, Wang Y, Cicuttini F, Davies-Tuck M, Hinman R. Higher dynamic medial knee load predicts greater cartilage loss over 12 months in medial knee osteoarthritis. Ann Rheum Dis. 2011;70:1770-4.

10. Bennell K, Hunt M, Wrigley T, Lim B, Hinman R. Role of muscle in the genesis and management of knee osteoarthritis. Rheum Dis Clin North Am. 2008;34:731-54.

11. Sharma L, Dunlop D, Cahue S, Song J, Hayes K. Quadriceps strength and osteoarthritis progression in malaligned and lax knees. Ann Internal Med. 2003;138:613-9.

12. Bukowski E, Conway A, Glentz L, Kurland K, Galantino M. The effect of iyengar yoga and strengthening exercises for people living with osteoarthritis of the knee: A case series. Int Q Community Health Educ. 2006;26:287-305.

13. Brown K, Koziol J, Lotz M. A yoga-based exercise program to reduce the risk of falls in seniors: A pilot and feasibility study. J Altern Complement Med. 2008;14:454-7.

14. Chen K, Chen M, Hong S, Chao H, Lin H, Li C. Physical fitness of older adults in senior activity centres after 24-week silver yoga exercises. J Clin Nurs. 2008;17:2634-46.

15. DiBenedetto M, Innes K, Taylor A, Rodeheaver P, Boxer J, Wright J, et al. Effect of a gentle Iyengar yoga program on gait in the elderly: An exploratory study. Arch Phys Med Rehabil. 2005;86:1830-7.

16. Kolansinski S, Garfinkel M, Gilden T, Matz W, VanDyke A, Schumacher H. Iyengar yoga for treating symptoms of osteoarthritis of the knees: A pilot study. J Altern Complement Med. 2005;11:689-93.

17. Ulger O, Yagli N. Effects of yoga on balance and gait properties in women with musculoskeletal problems: A pilot study. Complement Ther Clin Prac. 2011;17:13-5.

18. Felson D, Zhang Y, Hannan M, Naimark A, Weissman B, Aliabadi P, et al. The incidence and natural history of knee osteoarthritis in the elderly. The Framingham Osteoarthritis Study. Arthritis Rheum. 1995;38:1500-5.

19. Felson D, Zhang Y, Hannan M, Naimark A, Weissman B, Aliabadi P, et al. Risk factors for incident radiographic knee osteoarthritis in the elderly. The Framingham Study. Arthritis Rheum. 1997;40:728-33.

20. Oliveria S, Felson D, Reed J, Cirillo P, Walker A. Incidence of symptomatic hand, hip and knee osteoarthritis among patients in a health maintenance organization. Arthritis Rheum. 1995;38(8):1134-41.

21. Bennell K, Wrigley T, Hunt M, Lim B, Hinman R. Update on the role of muscle in the genesis and management of knee osteoarthritis. Rheum Dis Clin North Am. 2013;39:145-76.

22. Unruh A. Gender variations in clinical pain experience. Pain. 1996;65:123-67.

23. Felson D, Naimark A, Anderson J, Kazis L, Castelli W, Meenan R. The prevalence of knee osteoarthritis in the elderly. The Framingham Osteoarthritis Study. Arthritis Rheum. 1987;30:914-8.

24. Felson D, Zhang Y. An update on the epidemiology of knee and hip osteoarthritis with a view to prevention. Arthritis Rheum. 1998;41:1343-55.

25. Hochberg M, Kasper J, Williamson J, Skinner A, Fried L. The contribution of osteoarthritis to disability: Preliminary data from the Women's Health and Aging Study. J Rheumatol. 1995;22:16-8.

26. Gross M, Huffman G, Phillips C, Wray J. Intramachine and intermachine reliability of the Biodex and Cybex II for knee flexion and extension peak torque and angular work. JOSPT. 1991;13(6):329-35.

27. Bolton J, Wilkinson R. Responsiveness of pain scales: A comparison of three pain intensity measures in chiropractic patients. J Manipulative Physiol Ther. 1998;21:1.

28. Stratford P, Spadoni G. The reliability, consistency and clinical application of a numeric pain rating scale. Physiother Can. 2001;53:88-91.

29. Ferraz M, Quaresma M, Aquino L, Atra E, Tugwell P, Goldsmith C. Reliability of pain scales in the assessment of literate and illiterate patients with rheumatoid arthritis. J Rheumatol. 1990;17:1022.

30. Jensen M, Karoly P, Braver S. The measurement of clinical pain intensity: A comparison of six methods. Pain. 1986;27:117-26.

31. Jensen M, Turner J, Romano J, Fisher L. Comparative reliability and validity of chronic pain intensity measures. Pain. 1999;83:157-62.

32. Berthier F, Potel G, Leconte P, Touze M, Baron D. Comparative study of methods of measuring acute pain intensity in an ED. Am J Emerg Med. 1998;16:132-6.

33. Paradowski P, Bergman S, Sunden-Lundius A, Lohmander L, Roos E. Knee complaints vary with age and gender in the adult population. Population-based reference data for the Knee injury and Osteoarthritis Outcome Score (KOOS). BMC Musculoskeletal Disorders. 2006;7:38.

34. Roos E, Klassbo M, Lohmander L. WOMAC osteoarthritis index. Reliability, validity, and responsiveness in patients with arthroscopically assessed osteoarthritis. . Scand J Rheumatol. 1999;28:210-5.

35. Frobell R, Svensson E, Gothrick M, Roos E. Self-reported activity level and knee function in amateur football players: the influence of age, gender, history of knee injury and level of competition. . Knee Surg Sports Traumatol Arthrosc. 2008;16:713-9.

36. Ageberg E, Forssblad M, Herbertsson P, Roos E. Sex differences in patient-reported outcomes after anterior cruciate ligament reconstruction: data from the Swedish knee ligament register. Am J Sports Med. 2010;38:1334-42.

37. Alviar M, Olver J, Brand C, Hale T, Khan F. Do patient-reported outcome measures used in assessing outcomes in rehabilitation after hip and knee arthroplasty capture issues relevant to patients? Results of a systematic review and ICF linking process. . J Rehabil Med. 2011;43:374-81.

38. Collins N, Misra D, Felson D, Crossley K, Roos E. Measures of knee function: International Knee Documentation Committee (IKDC) Subjective Knee Evaluation Form, Knee Injury and Osteoarthritis Outcome Score (KOOS), Knee Injury and Osteoarthritis Outcome Score Physical Function Short Form (KOOS-PS), Knee Outcome Survey Activities of Daily Living Scale (KOS-ADL), Lysholm Knee Scoring Scale, Oxford Knee Score (OKS), Western Ontario and McMaster Universities Osteoarthritis Index (WOMAC), Activity Rating Scale (ARS), and Tegner Activity Score (TAS). . Arthritis Care Res. 2011;63:S208-S28.

39. Collins N, Roos E. Patient-reported outcoms for total hip and knee arthroplasty: Commonly used instruments and attributes of a "good" measure. Clin Geriatric Med. 2012;28:367-94.

40. Roos E, Roos H, Lohmander L. WOMAC Osteoarthritis Index - Additional dimensions for use in subjects with post-traumatic osteoarthritis of the knee. Osteoarthritis Cart. 1999;7:216-21.

41. Hawker G, Davis A, French M, Cibere J, Jordan J, March L, et al. Development and preliminary psychometric testing of a new OA pain measure - an OARSI/OMERACT initiative. Osteoarthritis Cart. 2008;16:409-14.

42. Hawker G, Stewart L, French M, Cibere J, Jordan J, March L, et al. Understanding the pain experience in hip and knee osteoarthritis - an OARSI/OMERACT initiative. Osteoarthritis Cart. 2008;16:415-22.

43. Davis A, Lohmander L, Wong R, Venkataramanan V, Hawker G. Evaluating the responsiveness of the ICOAP following hip or knee replacement. Osteoarthritis Cart. 2010;18:1043-5.

44. ATS. ATS Statement: Guidelines for the Six-Minute Walk test. Am J Respir Crit Care Med. 2002;166:111-7.

45. Cahalin L, Mathier M, Semigran M, Dec G, DiSalvo T. The Six-Minute Walk Test predicts peak oxygen uptake and survival in patients with advanced heart failure. Chest. 1996;110:325-32.

46. McCarthy E, Horvat M, Holtsberg P, Wisenbaker J. Repeated chair stands as a measure of lower limb strength in sexagenarian women. J Gerontol Biol Med Sci. 2004;59:1207-12.

47. Smith W, Del Rossi G, Adams J, Abderlarahman K, Asfour S, Roos B, et al. Simple equations to predict concentric lower-body muscle power in older adults using the 30-second chair-rise test: A pilot study. Clin Int Aging. 2010;5:173-80.

48. Blair S, Kohl H, Paffenbarger R, Clark D, Cooper K, Gibbons L. Physical fitness and all-cause mortality: A prospective study of healthy men and women. J Am Med Ass. 1989;22:2395-401.

49. Beekley M, Brechue W, deHoyos D, Garzarella L, Werber-Zion G, Pollock M. Cross-validation of the YMCA submaximal cycle ergometer test to predict VO2max. Res Q Exerc Sport. 2004;75:337-42.
