# Supplementary material for: A Yoga Strengthening Program Designed to Minimize the Knee Adduction Moment for Women with Knee Osteoarthritis: A Proof-Of-Principle Cohort Study
Source: PLoS One. 2015 Sep 14;10(9):e0136854. doi: 10.1371/journal.pone.0136854 (PMC4569287; doi:10.1371/journal.pone.0136854)
Supplement: S1 Appendix — (DOCX) [file pone.0136854.s003.docx]

**Warm-up**

The warm-up will include large body movements against the resistance of gravity only. Postural cues will be emphasized.

**Strengthening Series**

When performed properly, each of the exercises produces a small knee adduction moment, suggesting each yields a minimal load on the medial knee. Below are descriptions of the required exercises and suggestions for modification and progression. Progression is an important component of the program. The exercise instructor will be consulted regarding the modifications to ensure the participants are cued to their modification. A general rule will be to encourage progression every 2-3 weeks.

Participants will be encouraged to work at a rating of perceived exertion between 5 and 7. Participants will be asked to seek a modification if their pain increases by more than 2/10.

The following outlines the core strengthening exercises to be included in every class.

| **Exercise** | **Modification** | **Progression** | **Duration, Repetition** |
| --- | --- | --- | --- |
| 1. **Squat** | | | |
| 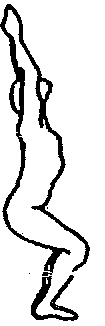  Level 4 | *Minor*: Change exercise to wall squats (removes balance challenge).  *Major*: Provide a chair and request participant to hover above the chair. | *Level 1*: Hands on hips, bend knees to 30°. | Hold 5 s. Repeat 2 times. |
|  |  | *Level 2*: Hands on hips, bend knees to 60°. | Hold 10 s. Repeat 2 times. |
|  |  | *Level 3*: Shoulders flexed to 90° with elbows straight, bend knees to 60°. | Hold 10 s. Repeat 5 times. |
|  |  | *Level 4*: Shoulders flexed to 180° (or as close as possible), bend knees to 80°. Look to the ceiling for an added balance challenge. | Hold 10 s. Repeat 5 times. |

| 1. **Squat – Wide-Legged** | | | |
| --- | --- | --- | --- |
| 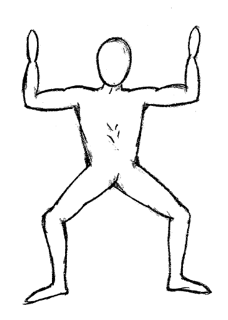  Level 3 | *Minor*: Reduce the width of stance. Change to wall squats.  *Major*: Provide a chair and request participant to hover above the chair. | *Level 1*: Hands on hips, bend knees to 30°. | Hold 5 s. Repeat 2 times. |
|  |  | *Level 2*: Hands on hips, bend knees to 60°. | Hold 10 s. Repeat 2 times. |
|  |  | *Level 3*: Shoulders abducted to 90° with elbows at 90°, bend knees to 60°. | Hold 10 s. Repeat 5 times. |
|  |  | *Level 4*: Shoulders flexed to 180° (or as close as possible), bend knees to 80°. Look to the ceiling for an added balance challenge. | Hold 10 s. Repeat 5 times. |
| 1. **Supported Lunge** | | | |
| 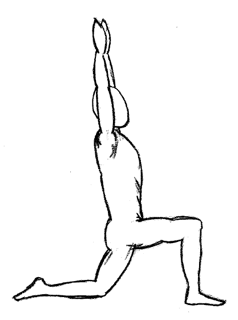  Level 4 | *Minor*: Provide a pillow beneath the trail leg (hip in extension). Reduce hip range of motion for the lead and trail legs.  *Major*: Kneeling position. | *Level 1*: Hands on hips, the hip of the trail leg is in neutral position (no flexion, no extension). | Hold 5 s. Repeat 2 times. |
|  |  | *Level 2*: Hands on hips, the hip of the trail leg is in extension. | Hold 10 s. Repeat 2 times. |
|  |  | *Level 3*: Shoulders flexed to 90° with elbows straight, the hip of the trail leg is in extension. | Hold 10 s. Repeat 3 times. |
|  |  | *Level 4*: Shoulders flexed to 180° (or as close as possible), the hip of the trail leg is in extension. | Hold 10 s. Repeat 3 times. |

| 1. **Lunge – Lateral Trunk** | | | |
| --- | --- | --- | --- |
| 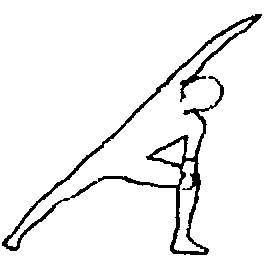  Level 2 | *Minor*: Provide support with a chair or block.  *Major*: Sitting on a chair, work towards the upper body position | *Level 1*: Place elbow on the knee of the lead leg. Place the other hand on the hip. | Hold 5 s. Repeat 2 times. |
|  |  | *Level 2*: Place elbow on the knee of the lead leg. Stretch the other shoulder such that it is flexed to 180° (or as close as possible). | Hold 10 s. Repeat 2 times. |
|  |  | *Level 3*: Place hand beside the foot instep of the lead leg. Stretch the other shoulder such that it is flexed to 180° (or as close as possible). | Hold 10 s. Repeat 3 times. |
|  |  | *Level 4*: Place hand beside the lateral side of foot, of the lead leg. Stretch the other shoulder such that it is flexed to 180° (or as close as possible). | Hold 10 s. Repeat 3 times. |
| 1. **Lunge – Upright Trunk 1** | | | |
| 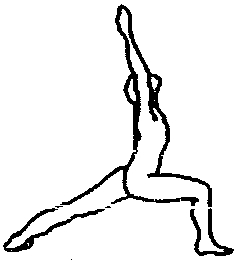  Level 4 | *Minor*: Reduce length of stride.  *Major*: Supported lunge. | *Level 1*: Hands on hips. | Hold 5 s. Repeat 2 times. |
|  |  | *Level 2*: Shoulders flexed to 90° with elbows straight. | Hold 10 s. Repeat 2 times. |
|  |  | *Level 3*: Shoulders flexed to 180° (or as close as possible.) | Hold 10 s. Repeat 3 times. |
|  |  | *Level 4*: Shoulders flexed to 180° (or as close as possible.) Look to the ceiling for an added balance challenge. | Hold 10 s. Repeat 3 times. |

| 1. **Lunge – Upright Trunk 2** | | | |
| --- | --- | --- | --- |
| 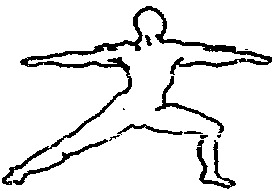  Level 2-4 | *Minor*: Reduce length of stride.  *Major*: Supported lunge. | *Level 1*: Hands on hips. | Hold 5 s. Repeat 2 times. |
|  |  | *Levels 2-4*: Arms abducted to 90° with elbows straight. | Hold 10 s. Repeat 2-3 times. |
| 1. **Bridge** | | | |
| 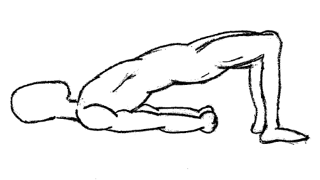Level 2-4 | *Minor*: Reduce height of bridge.  *Major*: Place a support (e.g., 4 inch foam block) under the sacrum for a passive stretch). | *Level 1*: Arms by side. | Hold 5 s. Repeat 2 times. |
|  |  | *Levels 2-4*: Clasp hands together. Increase height of bridge as appropriate. | Hold 10 s. Repeat 5 times. |

**Cool-Down**

Sitting or lying position for stretches of major muscle groups of the lower extremity:

- Trunk flexors, extensors and rotators,
- Hip flexors, extensors, adductors,
- Knee flexors and extensors, and
- Ankle plantarflexors.

Each class will focus on stretching the knee flexors and extensors.
