# Supplementary material for: A Yoga Strengthening Program Designed to Minimize the Knee Adduction Moment for Women with Knee Osteoarthritis: A Proof-Of-Principle Cohort Study
Source: PLoS One. 2015 Sep 14;10(9):e0136854. doi: 10.1371/journal.pone.0136854 (PMC4569287; doi:10.1371/journal.pone.0136854)
Supplement: S2 Appendix — (DOCX) [file pone.0136854.s004.docx]

**YMCA Cycle Ergometer Submaximal Test**

- Used to estimate VO_2_ max
- Uses 3 or more consecutive workloads of increasing resistance designed to raise HR to 85% of age-predicted max
- Pedaling rate is **50 rpm**
- Initial Workload is **25W**
- HR during *last minute of 1^st^ workload* determines the loading sequence for *subsequent workloads*

**Procedure**:

1. Estimate participant’s age-predicted HRmax (220-age), then calculate 85%.

Participant Age: ____________

220 – Age: ____________

85% of predicted max: ____________

1. Set the 1^st^ workload at 25W
2. Using HR monitor, take HR in 3^rd^ minute of 1^st^ Workload

HR during 3^rd^ Minute: ____________

1. Determine Subsequent Workloads:

| **1^st^ Workload** | 25W | | | |
| --- | --- | --- | --- | --- |
| *SUBSEQUENT:* | **HR <80bpm** | **HR 80-89bpm** | **HR 90-100bpm** | **HR >100bpm** |
| **2^nd^ Workload** | 125W | 100W | 75W | 50W |
| **3^rd^ Workload** | 150W | 125W | 100W | 75W |
| **4^th^ Workload** | 175W | 150W | 125W | 100W |
| **etc.** | *If additional workloads are required to achieve within 10bpm of 85% HRmax, add 25W to previous workload* | | | |

1. Measure the HR during last 15 seconds of minutes 2 and 3 at each workload. If these HR differ by more than 5bpm, extend the workload an extra minute until the HR stabilizes. The test is terminated when the participant’s steady-state HR is within **10 beats** of 85% HRmax.

| **Participant ID:** | | | | | **Date:** | | |
| --- | --- | --- | --- | --- | --- | --- | --- |
| **Age:** | | | **Gender: M** / **F** | | | **Body Mass:** | |
| **85% HRMax[(220-Age) x 0.85]:** | | | | | **Seat Height:** | | |
| **Time (min)** | | **Resistance (W)** | | **Cadence (rpm)** | **HR**  **(bpm)** | | **RPE** |
| **1^st^ Workload** | 1:00 |  | |  |  | |  |
|  | 2:00 |  | |  |  | |  |
|  | 3:00 |  | |  |  | |  |
|  | 4:00* |  | |  |  | |  |
| **2^nd^ Workload** | 1:00 |  | |  |  | |  |
|  | 2:00 |  | |  |  | |  |
|  | 3:00 |  | |  |  | |  |
|  | 4:00* |  | |  |  | |  |
| **3^rd^ Workload** | 1:00 |  | |  |  | |  |
|  | 2:00 |  | |  |  | |  |
|  | 3:00 |  | |  |  | |  |
|  | 4:00* |  | |  |  | |  |
| **4^th^ Workload** | 1:00 |  | |  |  | |  |
|  | 2:00 |  | |  |  | |  |
|  | 3:00 |  | |  |  | |  |
|  | 4:00* |  | |  |  | |  |
| **Recovery****  **(reduce resistance)** | 1:00 |  | |  |  | |  |
|  | 2:00 |  | |  |  | |  |
|  | 3:00 |  | |  |  | |  |
|  | 4:00 |  | |  |  | |  |
|  | 5:00 |  | |  |  | |  |

*4^th^ minute for each workload only required if HR during 2^nd^ and 3^rd^ minute are not at steady state (within 5bpm)

**An active recovery period of 2-5 minutes should immediately follow this test, reduce resistance and cadence

Equation to Predict VO_2_max for YMCA Cycle Ergometer Test:

1. **Determine Power Output:**

- SM_1_ = sub-maximal VO_2_ at second-last workload
- SM_2_ = sub-maximal VO_2_ at last workload

| VO_2_ = | Workload (W) | x 10.8 | + 3.5 +3.5 |
| --- | --- | --- | --- |
|  | Body Mass (kg) |  |  |

| SM_1_ = | ____________ | x 10.8 | + 3.5 +3.5 |
| --- | --- | --- | --- |
|  |  |  |  |
|  |  |  |  |
| = | **_____________** |  |  |

| SM_2_ = | ____________ | x 10.8 | + 3.5 +3.5 |
| --- | --- | --- | --- |
|  |  |  |  |
|  |  |  |  |
| = | **_____________** |  |  |

1. **Determine slope of the line of best fit:**

| b_2_ = | SM_2_ – SM_1_ |
| --- | --- |
|  | HR_2_ – HR_1_ |

| b_2_ = | ____ – ____ |
| --- | --- |
|  | – |

| b_2_ = |  |
| --- | --- |
|  | __________ |

1. Determine VO_2_max

VO_2_max = SM_2_ + [*b* x (HRmax – HR_2_)]

= ______ + [ ______ x ( ______ – ______ )]

VO_2_max =
